# Perioperative Risks and Long-Term Outcomes of Bilateral Adrenalectomy: A Multicenter Retrospective Cohort Study Across Cushing’s Syndrome and Non-Cushing Etiologies

**DOI:** 10.3390/medicina62071373

**Published:** 2026-07-17

**Authors:** Gökçen Güngör Semiz, Nusret Yılmaz, Mustafa Aydemir, Özlem Soyluk Selçukbiricik, Banu Şarer Yürekli, Hatice Özışık, Süheyla Görar, Güzin Fidan Yaylalı, Eda Ertörer, Hatice Öner, Selin Genç, Mehmet Emin Arayici, Şeyhmus Abakay, Mehmet Çağrı Ünal, Abdurrahman Çömlekçi, Serkan Yener, Tevfik Demir

**Affiliations:** 1 Division of Endocrinology and Metabolism, Department of Internal Medicine, Faculty of Medicine, Dokuz Eylul University, Izmir 35340, Turkey; gokcen.gungorsemiz@deu.edu.tr (G.G.S.); mehmetcagri.unal@deu.edu.tr (M.Ç.Ü.); comlekci@deu.edu.tr (A.Ç.); serkanyenerendokrin@gmail.com (S.Y.); drtevfikdemir@gmail.com (T.D.); 2 Division of Endocrinology and Metabolism, Department of Internal Medicine, Faculty of Medicine, Akdeniz University, Antalya 07070, Turkey; nusretyilmaz@akdeniz.edu.tr (N.Y.); aydemirmustafa@akdeniz.edu.tr (M.A.); 3 Division of Endocrinology and Metabolism, Department of Internal Medicine, Faculty of Medicine, İstanbul University, Istanbul 34093, Turkey; ozlem.soylukselcukbiricik@istanbul.edu.tr; 4 Division of Endocrinology and Metabolism, Department of Internal Medicine, Faculty of Medicine, Ege University, Izmir 35040, Turkey; banu.yurekli@ege.edu.tr (B.Ş.Y.); hatice.ozisik@ege.edu.tr (H.Ö.); 5 Division of Endocrinology, Department of Internal Medicine, Antalya Training and Research Hospital, Antalya 07100, Turkey; suheyla.gorar@sbu.edu.tr; 6 Division of Endocrinology and Metabolism, Department of Internal Medicine, Faculty of Medicine, Pamukkale University, Denizli 20070, Turkey; gyaylali@pau.edu.tr; 7 Department of Endocrinology and Metabolism, Adana Hospital, Başkent University School of Medicine, Adana 01120, Turkey; meertorer@baskent.edu.tr; 8 Department of Endocrinology and Metabolism, Marmara University Pendik Training and Research Hospital, Istanbul 34899, Turkey; hatice.oner@marmara.edu.tr; 9 Department of Endocrinology and Metabolism, Turgut Ozal Medical Center, Faculty of Medicine, Inonu University, Malatya 44280, Turkey; selin.genc@inonu.edu.tr; 10 Department of Biostatistics and Medical Informatics, Faculty of Medicine, Dokuz Eylül University, Izmir 35340, Turkey; mehmetemin.arayici@deu.edu.tr

**Keywords:** bilateral adrenalectomy, Cushing’s syndrome, survival analysis, adrenal crisis, mortality

## Abstract

*Background and Objectives:* Bilateral adrenalectomy (BADx) is a definitive treatment for diverse adrenal disorders, though data across etiologies remain limited. This multicenter study evaluated perioperative risks, long-term survival, and shifts in comorbidities in patients undergoing BADx for Cushing’s syndrome (CS) and non-CS indications. *Materials and Methods:* We retrospectively analyzed 103 adult patients (CS, *n* = 52; non-CS, *n* = 51) from 9 tertiary centers. Primary outcomes included early (≤30 days) and late mortality, complications (adrenal crisis, Nelson’s syndrome), and changes in comorbidity profiles. *Results:* The cohort (mean age 50.59 ± 13.82 years) had a median follow-up of 96 months. Early mortality occurred exclusively in CS patients (5.8%), while late mortality was comparable between groups. Overall 5-year survival rates were 83.3% for CS and 89.9% for non-CS (*p* = 0.360), with no significant differences across CS etiologies (*p* = 0.760). Adrenal crisis was significantly more frequent in CS (23% vs. 7%, *p* = 0.023). Nelson’s syndrome developed in 28% of Cushing’s disease patients. Postoperatively, both cohorts showed increased reliance on psychiatric and cardiovascular medications. Notably, osteoporosis rates were significantly higher than preoperative levels exclusively in the non-CS group (*p* = 0.006), highlighting the impact of long-term glucocorticoid replacement in this cohort. Two patients with congenital adrenal hyperplasia demonstrated remarkable long-term survival (8 and 30 years) across different surgical timings. *Conclusions:* BADx delivers rapid systemic remission and definitive control across a range of adrenal pathologies. However, the primary cause and the timing of surgery significantly impact the clinical course and the risk of complications. The resulting state of irreversible adrenal insufficiency requires comprehensive, lifelong surveillance and tailored hormone replacement.

## 1. Introduction

Bilateral adrenalectomy (BADx) is a definitive surgical intervention indicated for a diverse range of disorders, including Cushing’s disease (CD), ectopic Cushing’s syndrome (ECS), ACTH-Independent Macronodular Adrenal Hyperplasia (AIMAH), hereditary bilateral pheochromocytoma, congenital adrenal hyperplasia (CAH) refractory to medical management, and adrenal metastases from extra-adrenal malignancies [1,2]. Potent steroidogenesis inhibitors and precise radiotherapy techniques have emerged in the 21st century. Despite these advances, BADx remains an essential “ultima ratio” for patients with refractory hypercortisolism or life-threatening acute presentations where rapid biochemical control is mandatory [3]. While BADx provides rapid biochemical control of hormonal excess, it inevitably results in permanent primary adrenal insufficiency. This iatrogenic dependency necessitates lifelong glucocorticoid and mineralocorticoid replacement, leaving patients vulnerable to life-threatening adrenal crises during physiological stress [4].

In the specific context of Cushing’s disease, Nelson’s syndrome remains a critical long-term complication. Characterized by progressive pituitary tumor expansion and extreme Adrenocorticotropic hormone (ACTH) elevation due to the loss of glucocorticoid feedback, Nelson’s syndrome shows significant variability in prevalence and time to onset. This underscores the absolute necessity for lifelong radiological and biochemical surveillance, ideally using modern high-resolution MRI and serial ACTH monitoring [5].

Beyond survival, the clinical utility of BADx is often measured by its impact on cardiometabolic comorbidities. Landmark reviews highlight that biochemical control of hypercortisolism does not always equate to a complete reversal of clinical morbidities. Consequently, patients may continue to face a persistent metabolic and psychological burden long after successful surgery. While the resolution of cortisol or catecholamine excess typically improves arterial hypertension and glycemic control, the long-term magnitude and durability of these improvements across different etiological subgroups remain incompletely characterized in daily clinical practice. Most existing literature is limited to single-center experiences or focuses exclusively on Cushing’s syndrome, often lacking other etiologies [6]. To address this gap, we conducted a multicenter retrospective study. Our aim was to provide robust real-world data on the long-term clinical trajectories, comorbidity shifts, and outcomes of patients undergoing BADx for any indication.

## 2. Materials and Methods

Approval for this multicenter, retrospective cohort study was obtained from the Non-Interventional Research Ethics Committee of Dokuz Eylül University Faculty of Medicine (Decision No: 2024/10-11—Approval Date: 13 March 2024). The study involved 9 tertiary care centers. To ensure a representative sample and minimize selection bias, all consecutive adult patients (aged ≥18 years) who underwent bilateral adrenalectomy (BADx) for any clinical indication between 2000 and 2023 and were followed at the participating tertiary endocrinology clinics were retrospectively identified. Patients eligible for the study had to be in a confirmed postoperative state of complete bilateral adrenalectomy (BADx), either through a simultaneous or staged approach, with essential baseline demographic and clinical data available. Those who had unilateral adrenalectomy without further contralateral intervention during the study period, or who lacked verified diagnostic documentation, were excluded. For the 17 patients who underwent a staged surgical approach, the date of the completion of adrenalectomy (second surgery) was defined as the baseline for all survival and outcome analyses. Data was collected through a retrospective review of hospital electronic medical records. Two separate data collection forms were employed for CS and non-CS etiologies.

The primary outcomes included early mortality (≤30 days), late mortality, and long-term complications like adrenal crisis and Nelson’s Syndrome. Adrenal crisis was defined as acute clinical deterioration requiring parenteral glucocorticoid therapy. In the CS group, Nelson’s Syndrome was defined as radiological evidence of progressive pituitary tumor expansion and high ACTH concentrations. Changes in cardiometabolic comorbidities were assessed by comparing preoperative and postoperative medication requirements and clinical status. Assessment of proximal myopathy was based on documented patient self-reports at the time of clinical evaluation.

### Statistical Analysis

Statistical analyses were performed using SPSS (Version 29; IBM Corp., Armonk, NY, USA) and JAMOVI (version 2.7.4; jamovi project, Sydney, Australia). Kaplan–Meier survival curves and log-rank tests were generated in Python (version 3.10.12; Python Software Foundation, Beaverton, OR, USA) using the open-source libraries lifelines (version 0.30.0; open-source Python package, Cameron Davidson-Pilon, Canada) and matplotlib (version 3.10.9; Matplotlib Development Team/NumFOCUS, Austin, TX, USA). The flowchart was created using the grid package in R statistical software (version 4.2.0; R Foundation for Statistical Computing, Vienna, Austria; R Core Team, 2022). The normality of the data distribution was assessed using the Kolmogorov–Smirnov or Shapiro–Wilk tests. Continuous variables are presented as mean ± standard deviation (SD) for normally distributed data, or as median (interquartile range (IQR)) for non-normally distributed data. Categorical variables are expressed as frequencies and percentages (*n*, %). For comparisons between the Cushing’s and non-Cushing’s subgroups, the Independent Samples *t*-test was used for normally distributed continuous variables, and the Mann–Whitney U test was used for non-normally distributed variables. Categorical data were compared using the Chi-square test. The incidence of early and late mortality was calculated. Survival analysis was performed using the Kaplan–Meier method to estimate survival probabilities, with the restricted mean survival time (RMSE) used as a summary measure when the median survival is not estimable. Group differences in survival distributions were assessed using the log-rank test. Incidence rates were calculated by dividing the number of clinical events by the total person-time at risk, expressed as person-years of follow-up, and stratified by predefined time intervals. For each participant, person-time was accumulated from the date of bilateral adrenalectomy until the occurrence of the specific event (death) or the last follow-up visit. All analyses were conducted at a two-sided significance level of α = 0.05.

Missing retrospective data were handled using a complete-case analysis. Patients with missing parameters for specific comorbidity shifts or biochemical assessments were excluded only from those respective sub-analyses. This strategy preserved the maximum available cohort size for the primary endpoints. Additionally, a sensitivity analysis showed no significant differences in baseline clinical profiles between patients with complete and incomplete records. Finally, because sub-analyses within this rare-disease cohort were exploratory and hypothesis-driven, universal alpha-corrections such as the Bonferroni correction were not applied to avoid increasing Type II error rates. Instead, all exact *p*-values are reported transparently.

## 3. Results

### 3.1. Demographic Characteristics and Etiological Distribution

A total of 103 patients from nine different centers were included in this multicenter study. Seventeen patients underwent initial unilateral adrenalectomy. The clinical indications for the 17 patients who underwent staged surgery included pheochromocytoma, adrenal metastases, and AIMAH. For all remaining patients, BADx was performed as a single-stage procedure.

The study population consisted of 59 (57.3%) females and 44 (42.7%) males. The mean age of the participants at the time of study inclusion was 50.65 ± 13.77 years (range: 18–84), while the mean age at the time of bilateral adrenalectomy (BADx) was 40.29 ± 15.78 years. The median follow-up duration after BADx was 96 (0–444) months. Regarding the surgical approach, laparoscopic surgery was performed in 61.2% (*n* = 63) of patients, while open surgery was performed in 36.4% (*n* = 36). Procedures were reported according to the final surgical approach; however, specific data on conversions from laparoscopic to open surgery were unavailable due to the database’s retrospective nature. Of the 103 patients included in the study, 52 (50.5%) underwent BADx due to Cushing’s syndrome (CS), while 51 (49.5%) underwent the procedure for other clinical indications. Within the CS cohort, the underlying etiologies were distributed as follows: 25 patients (48.1%) with Cushing’s disease (CD), 12 (23.1%) with ECS, 12 (23.1%) with AIMAH, and 3 (5.7%) with primary pigmented nodular adrenocortical disease (PPNAD). Regarding the clinical indications within the non-CS group (*n* = 51), the most frequent etiology was pheochromocytoma (*n* = 39), followed by bilateral adrenal metastasis (*n* = 9), congenital adrenal hyperplasia (CAH) (*n* = 2), and tuberculosis (*n* = 1). The distribution of patients who underwent bilateral adrenalectomy by etiology is summarized in Table 1. Detailed demographic data and the distribution of comorbidities in all groups and across the two study groups (Cushing’s and non-Cushing’s) are presented in Table 2.

Regarding demographic and clinical characteristics, the proportion of female patients was significantly higher in the CS group compared to the non-CS cohort (*p* = 0.038). The body mass index (BMI) was also found to be markedly elevated in patients with Cushing’s syndrome (*p* < 0.001). In terms of surgical approach, laparoscopic adrenalectomy was performed at a significantly higher rate in the Cushing’s group (*p* = 0.010). Furthermore, the prevalence of comorbid conditions, including diabetes mellitus, hypertension, osteoporosis, and proximal myopathy, was significantly more frequent in the Cushing’s group than in the non-Cushing’s group (*p* = 0.001, *p* = 0.002, *p* < 0.001, and *p* < 0.001, respectively).

### 3.2. Preoperative Treatments and Interventions

The preoperative clinical management of the CS cohort (*n* = 52) involved multi-modal treatment strategies tailored to the specific etiology. In patients with Cushing’s disease specifically, pituitary surgery, radiotherapy, and medical therapy were administered either as monotherapy or in combination prior to BADx; however, a small number of patients (*n* = 4) underwent direct bilateral adrenalectomy. Similarly, in patients with ectopic Cushing’s syndrome, medical therapy was administered before proceeding to BADx; specifically, six patients received preoperative medical treatment to mitigate severe hypercortisolemia. Among the 12 patients with Ectopic Cushing’s Syndrome (ECS), the primary tumor was localized in four cases. These consisted of two pancreatic neuroendocrine tumors, one lung carcinoid, and one thymic carcinoid tumor. In the remaining eight patients, the primary focus remained unidentified. Medical stabilization for hypercortisolemia was achieved using ketoconazole, pasireotide, metyrapone, or octreotide. Notably, cabergoline was administered as an adjunctive therapy in four patients in combination with either ketoconazole or pasireotide.

**Figure 1:**
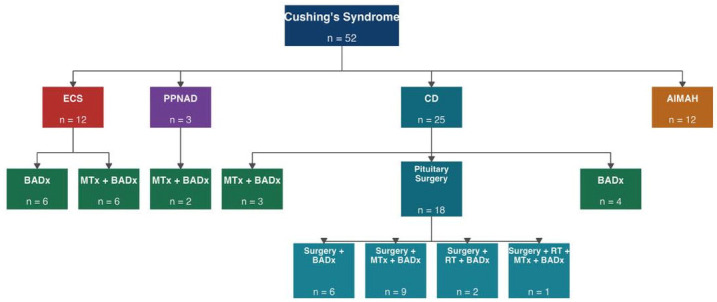
Comprehensive breakdown of preoperative treatment strategies across distinct Cushing’s syndrome etiologies. ECS, ectopic Cushing’s syndrome; PPNAD, primary pigmented nodular adrenocortical disease; CD, Cushing’s disease; AIMAH, ACTH-independent macronodular adrenal hyperplasia; BADx, bilateral adrenalectomy; MTx, medical therapy; RT, radiotherapy.

Within the non-Cushing’s cohort (*n* = 51), patients with adrenal metastases were evaluated with regard to their primary tumor origins. Among this subgroup, two patients received systemic chemotherapy before proceeding to BADx. Analysis identified renal cell carcinoma as the predominant primary malignancy (*n* = 7), followed by lung (*n* = 1) and thymic (*n* = 1) carcinomas. The preoperative treatment strategies specific to each Cushing’s syndrome subgroup are detailed in Figure 1.

### 3.3. Mortality Outcomes

Regarding the mortality data, 3 patients (2.9%) died during the early postoperative period (within the first 30 days), while 13 patients (12.6%) died during the long-term follow-up. Early mortality (within the first month) occurred exclusively in the Cushing’s group (5.8%, *n* = 3). These cases were distributed across three separate centers. Notably, one death occurred intraoperatively due to severe hemorrhage, while the remaining two occurred postoperatively within the first month due to thromboembolic events. Late mortality causes included acute myocardial infarction (MI), sepsis, adrenal crisis, and progression of the primary tumor. In five cases, the cause of death remained unknown. While all patients with early mortality were within the CS cohort, those with late mortality consisted of 7 patients from the CS group and 6 from the non-CS group. Data regarding mortality and long-term complications (adrenal crisis, Nelson’s syndrome) are presented in Table 3. The specific causes of early and late mortality, categorized by the underlying primary etiology, are presented in Table 4. When analyzing mortality rates by specific etiology, they were 20% (5/25) for Cushing’s disease (CD), 16% (2/12) for ECS, and 25% (3/12) for AIMAH. The mortality rate was substantially higher at 55% (5/9) for patients with bilateral adrenal metastases, while it was notably low at 2% (1/39) for those with pheochromocytoma.

### 3.4. Long-Term Complications and Clinical Follow-Up

In terms of long-term complications, the incidence of hospital admissions due to adrenal crisis was significantly greater in patients with CS (*p* = 0.023). Additionally, Nelson’s syndrome developed in 28% (*n* = 7) of the 25 patients diagnosed with Cushing’s disease. In patients who developed Nelson’s syndrome, the median interval from bilateral adrenalectomy to diagnosis was 96 months (range: 24–264). At the time of diagnosis, the median plasma ACTH level in these patients was 445 pg/mL (range: 231–1250).

The median follow-up duration (time since BADx) was 80.5 months (range: 0–444) in the CS group and 120 months (range: 2–360) in the non-CS group. Intra-group comparisons revealed notable shifts in comorbid conditions for both cohorts. Within the CS group, body mass index (BMI) decreased significantly (*p* < 0.001), and the prevalence of proximal myopathy decreased significantly (*p* < 0.001). Furthermore, a reduction in antihypertensive therapy requirements was observed in both the CS (*p* = 0.057) and non-CS (*p* = 0.092) cohort.

Notably, both cohorts exhibited an increased reliance on psychiatric, cardiovascular, and osteoporosis medications during the follow-up period. Regarding bone health, while the proportion of patients receiving pharmacological treatment for osteoporosis showed a numerical upward trend in both groups, this longitudinal shift reached statistical significance only in the non-CS cohort (*p* = 0.006), whereas the increase in the CS group did not. Among the patients who developed osteoporosis in the non-CS group, 5 were female and 6 were male. Within these female patients, only one was postmenopausal, while the remaining 4 were premenopausal (aged 25–48 years). Detailed changes in comorbidities following BADx in both cohorts are presented in Table 5.

Corticosteroid replacement preparations used by patients included hydrocortisone, prednisolone, methylprednisolone, and dexamethasone. Notably, prednisolone was the most frequently prescribed agent in both cohorts. The median body surface area-adjusted hydrocortisone dose was 11.50 (range: 5.49–20.13) mg/m^2^ in the CS group and 12.38 (range: 6.50–30.86) mg/m^2^ in the non-CS group. When comparing corticosteroid regimens by daily hydrocortisone dose per body surface area (mg/m^2^), no statistically significant difference was observed between the two groups (*p* = 0.111).

Regarding clinical outcomes, remission was achieved in 49 of 52 patients (94.2%) in the CS cohort; follow-up data are currently unavailable for 3 patients. In the pheochromocytoma subgroup (*n* = 39), only one patient had a recurrence during follow-up. Furthermore, among the nine patients with malignancy and bilateral adrenal metastases, disease progression was identified in two individuals (22.2%).

### 3.5. Survival Outcomes

**Figure 2:**
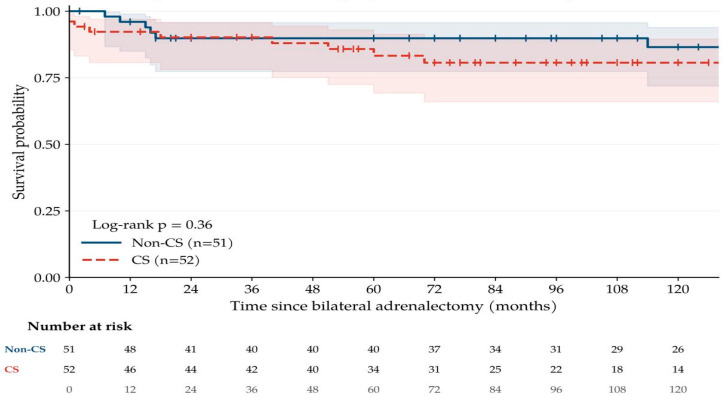
Comparison of long-term survival outcomes between patients undergoing bilateral adrenalectomy for Cushing’s syndrome (CS, *n* = 52) and non-Cushing’s syndrome indications (non-CS, *n* = 51). The blue and red shaded areas represent the 95% confidence intervals for the Non-CS and CS groups, respectively.

Survival did not differ significantly between the CS (*n* = 52) and non-CS (*n* = 51) groups (Figure 2). Over 12,737 person-months of follow-up, 16 events occurred. Median survival was not reached in either group. Five-year survival was 83.3% in the CS group versus 89.9% in the non-CS group, and the restricted mean survival time was numerically lower in the CS group (344.7 vs. 389.4 months). The difference was not statistically significant (log-rank *p* = 0.36).

**Figure 3:**
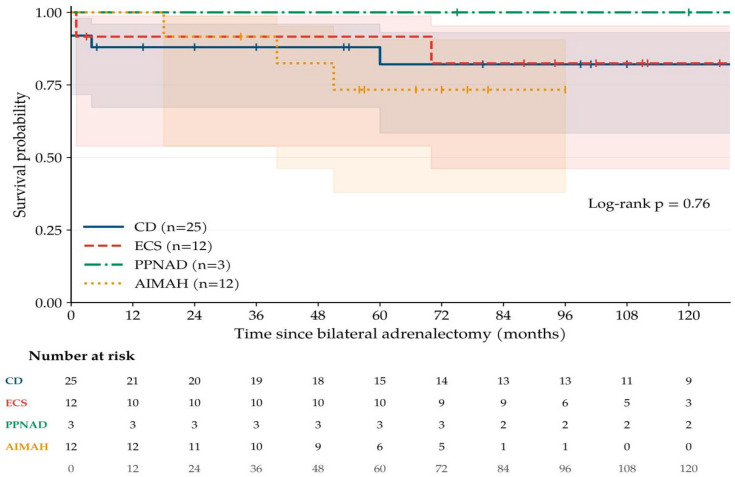
Comparison of long-term survival outcomes across the four distinct Cushing’s syndrome etiologies. The cohorts consist of Cushing’s Disease (CD, *n* = 25), Ectopic Cushing’s Syndrome (ECS, *n* = 12), Primary Pigmented Nodular Adrenocortical Disease (PPNAD, *n* = 3), and ACTH-Independent Macronodular Adrenal Hyperplasia (AIMAH, *n* = 12). The blue, red, green, and yellow shaded areas represent the 95% confidence intervals for the CD, ECS, PPNAD, and AIMAH groups, respectively.

Survival also did not differ across the four CS etiologies (Figure 3): Cushing’s disease (*n* = 25), ECS (*n* = 12), PPNAD (*n* = 3), and AIMAH (*n* = 12). Over 6,080 person-months, 10 events occurred (incidence rate 0.16 per 100 months; 95% CI 0.08–0.30). Median survival was not reached in any group. Five-year survival ranged from 73.3% (AIMAH) to 100% (PPNAD), and restricted mean survival times were comparable (335–444 months). Differences were not significant (log-rank *p* = 0.76).

## 4. Discussion

Bilateral adrenalectomy (BADx) remains a definitive and indispensable intervention for immediate hormonal control, despite the rise in pharmacological and radiotherapeutic alternatives [3]. While existing literature extensively covers BADx in the context of hypercortisolism, data regarding postoperative morbidity and mortality in these patients remain limited [1,6,7,8,9]. More importantly, there is a significant lack of research on the long-term prognosis of patients undergoing BADx for non-CS indications. To address this gap, our study provides a comprehensive comparison between CS and other clinical etiologies. We evaluated morbidity, mortality, and long-term complications across these two distinct patient populations.

Our cohort showed distinct baseline differences: the CS group exhibited a higher female predominance (*p* = 0.038) and a significantly higher mean BMI (*p* < 0.001) than the non-CS group, consistent with the classic clinical profile of hypercortisolism. Age at BADx was comparable between groups, ensuring a balanced baseline for outcome analysis. Preoperatively, comorbidities, including diabetes, hypertension, osteoporosis, and proximal myopathy, were significantly more prevalent in the Cushing’s cohort (*p* = 0.001, *p* = 0.002, *p* < 0.001, *p* < 0.001, respectively). In contrast, the non-CS group had a higher prevalence of malignancy. These findings reflect the divergent systemic burdens of the two etiologies. Specifically, the CS group was characterized by a metabolic burden, while the non-CS group faced an oncological burden.

Regarding surgical technique, laparoscopic BADx was performed in 61.2% of our cohort. While this technique is the gold standard due to reduced morbidity and faster recovery [3,10], our rates were slightly lower than those reported by Szabo et al. (68%) and Nagendra et al. (100%) [8,9]. However, subgroup analysis revealed an etiological divergence: the laparoscopy rate was significantly higher in the CS group (76.9%) than in the non-CS group (51.0%; *p* = 0.010). This distinction is crucial, as most of the literature focuses exclusively on Cushing’s syndrome, and our results align with global data. The lower laparoscopic rate in the non-Cushing’s cohort is likely due to the inclusion of bilateral adrenal metastases requiring oncological clearance via open surgery and infant CAH cases.

Early mortality (within the first month) occurred exclusively in the CS group (5.8%, *n* = 3), primarily due to intraoperative hemorrhage and thromboembolic events. While overall mortality was higher in the CS cohort than in the non-CS group (19.2% vs. 11.8%), this difference did not reach statistical significance (*p* = 0.296), and no significant differences were observed in age at mortality or in the duration from surgery to death. However, 66.7% of all first-year deaths occurred in patients with Cushing’s syndrome. This underscores the severe perioperative and long-term risks driven by chronic hypercortisolism-induced complications, such as hypercoagulability, obesity, and pre-existing cardiovascular burdens. Our early mortality rates are largely consistent with the literature, which reports rates ranging from 3% to 15.15% [1,7,8,11]. However, subgroup analysis revealed an etiological divergence: overall mortality was 20% in CD, 16% in ECS, and 25% in AIMAH. Our ECS mortality was notably lower than reported by Ejaz et al. (62.8%) and Morris et al. (59.2%) [12,13]. This divergence likely stems from the timing of BADx and from our high proportion of occult cases [8,12], which may carry a better prognosis than aggressive malignancies [13]. However, this finding must be interpreted with caution due to the limited sample size of this specific subgroup (*n* = 12). This small cohort may lack sufficient statistical power to reflect the true long-term mortality risk typically associated with severe hypercortisolism. Nevertheless, the combination of occult disease and early surgical intervention likely enhanced outcomes in our series. Surgical resection remains the first-line treatment for localized ECS, and BADx serves as a definitive intervention in occult cases, enabling prompt biochemical control of severe hypercortisolemia [12,14]. Although the mortality rate in the ECS group appeared lower than in the CD group, no statistically significant difference in overall survival was observed among the Cushing’s syndrome subgroups (*p* = 0.760).

Following BADx, permanent adrenal insufficiency necessitates lifelong replacement therapy, posing a constant risk of adrenal crisis. In our cohort, the overall adrenal crisis rate [15.5%] was lower than the 21–63% reported in the literature [7,8,15]. However, crises were significantly more frequent in the CS group compared to the non-CS group (23% vs. 7%; *p* = 0.023), likely due to prolonged HPA-axis suppression. Mortality from adrenal crisis remained rare, occurring in only one patient (operated bilateral pheochromocytoma). Furthermore, Nelson’s syndrome—a critical long-term complication unique to Cushing’s disease—developed in 28% (7/25) of our patients at a median of 96 months (range: 24–264 months). These findings closely align with the 21–38% prevalence reported in major series [1,5,7,8] and underscore the vital importance of lifelong hormonal and radiological surveillance in this population.

Post-BADx, the CS group showed significant clinical recovery, including marked weight loss (*p* < 0.001) and regression of proximal myopathy (*p* < 0.001). These outcomes are consistent with the existing literature [1,8]. Antihypertensive therapy requirements showed a downward trend in both groups (CS: *p* = 0.057; non-CS: *p* = 0.092). However, a notable finding was the increased long-term reliance on psychiatric, cardiovascular, and osteoporosis medications across the entire cohort. Psychiatric symptom persistence in our series aligns with the limited regression reported in previous studies [7,8]. Despite achieving biochemical cure in patients with CS, BADx patients often maintain a lower quality of life and higher anxiety or depression scores compared to those treated with pituitary surgery or medication [16]. Our findings reinforce this trend through the documented increase in psychiatric medication use in both cohorts. For non-CS patients, transitioning to lifelong hormone dependency likely drives these psychological burdens even without preoperative hypercortisolemia. Backed by our extended follow-up (median 96 months), these persistent challenges emphasize that biochemical cure does not equate to complete clinical resolution.

The rise in osteoporosis prevalence was statistically significant exclusively within the non-CS group (*p* = 0.006). Importantly, the rise in osteoporosis within the non-CS cohort was independent of sex or menopausal status. The nearly equal sex distribution and the predominance of young, premenopausal females support this finding. These results reinforce lifelong iatrogenic glucocorticoid replacement as the primary driver of long-term osteoporosis in this group. This risk appears distinct from natural, age- or sex-related bone loss. Larger prospective studies are warranted to further optimize these management strategies.

A notable highlight of our cohort is the long-term survival of two patients with congenital adrenal hyperplasia (CAH), providing rare insights into their individual clinical courses. Despite being diagnosed at age one, the first patient underwent BADx and had a 30-year follow-up. In contrast, the second patient underwent surgery at age 22 with an 8-year follow-up. A 2018 meta-analysis supports BADx as a safe alternative for CAH patients refractory to medical management [17]. However, it recommends delaying surgery until adulthood to mitigate the risks of adrenal crisis and ectopic adrenal rest tumors. In this regard, our cases contribute to the literature by providing rare longitudinal evidence of clinical stability across different surgical timing points. These findings underscore the feasibility of BADx in refractory CAH when maintained with lifelong care.

The primary strength of this study lies in its multicenter design across 9 tertiary centers, which provides robust real-world data across a broad spectrum of clinical indications. Notably, our cohort has a median follow-up of 96 months. This timeline is one of the longest reported in the literature for such a diverse etiological population, significantly enhancing the clinical value of our series. This prolonged longitudinal perspective helps identify late-onset metabolic burdens, including a significant rise in osteoporosis. These critical long-term impacts of bilateral adrenalectomy might otherwise remain undetected in shorter follow-up periods.

### Study Limitations

Our study has several limitations regarding its internal and external validity. Inherently, its retrospective nature restricts data standardization and introduces potential selection and information biases due to missing clinical parameters, such as specific conversion rates from laparoscopic to open surgery. The prolonged historical follow-up period across nine distinct tertiary centers may also introduce potential heterogeneities in surgical techniques, perioperative management, and the sensitivity of hormonal assays over time. Furthermore, the limited number of recorded survival events (*n* = 16) constrained the statistical power to detect minor subgroup differences. This limitation increased the risk of Type II error, as reflected in the survival model’s modest discriminative capacity (Concordance: 0.563). Furthermore, the representativeness of specific etiological subgroups is a clear constraint. The small sample sizes for rare conditions like PPNAD (*n* = 3) and CAH (*n* = 2) preclude reliable, generalizable conclusions, thereby limiting the universal applicability of our secondary outcomes. Similarly, the inclusion of adrenal metastases (*n* = 9) inevitably impacts the overall cohort due to their high baseline mortality. To prevent a distorted clinical picture, we strictly avoided a pooled cohort approach. Instead, we performed stratified survival analyses by separating patients into distinct CS and non-CS groups, alongside further etiological sub-analyses within the Cushing’s cohort. Additionally, the assessment of proximal myopathy and psychological burdens relied on documented clinical reports and patient self-reports rather than standardized prospective scoring tools, which may be subject to underreporting. Despite these constraints, this cohort remains one of the largest and most comprehensive series evaluating long-term outcomes of bilateral adrenalectomy across such a wide etiological spectrum.

## 5. Conclusions

In conclusion, bilateral adrenalectomy is a highly effective definitive treatment for refractory Cushing’s syndrome and heterogeneous adrenal pathologies, providing immediate biochemical control and significant comorbidity regression. However, long-term survival trajectories and complication profiles remain distinct across different etiological subgroups. Transitioning to permanent adrenal insufficiency necessitates lifelong multidisciplinary surveillance. This continuous monitoring is essential to manage persistent cardiovascular burdens, psychiatric issues, and potential complications like Nelson’s syndrome. Notably, the significant rise in postoperative osteoporosis is particularly evident in non-CS patients. This trend underscores the need for precise glucocorticoid dose optimization and continuous monitoring. These proactive measures are essential to mitigate iatrogenic risks and ensure favorable clinical outcomes.

## Figures and Tables

**Table 1:** Etiological Distribution of Patients Undergoing Bilateral Adrenalectomy (*n* = 103).

Etiology	Number of Patients (*n*)	Percentage (%)
1. Cushing’s Syndrome Group	52	50.5%
- Cushing’s Disease	25	24.3%
- AIMAH (ACTH-independent Macronodular Adrenal Hyperplasia)	12	11.6%
- Ectopic Cushing’s Syndrome	12	11.6%
- PPNAD (Primary Pigmented Nodular Adrenocortical Disease)	3	2.9%
2. Non-Cushing’s İndications Group	51	49.5%
- Pheochromocytoma	39	37.9%
- Bilateral Adrenal Metastases	9	8.7%
- Congenital Adrenal Hyperplasia (CAH)	2	1.9%
- Tuberculosis-related Adrenal Involvement	1	1.0%
Total	103	100%

**Table 2:** Demographic and Clinical Characteristics of the Patient Cohort (Before BADx).

Variable	Total (*n* = 103)	Cushing’s Group (*n* = 52)	Non-Cushing’s Group (*n* = 51)	*p*-Value
Age (years), Mean ± SD	50.65 ± 13.77	52.05 ± 12.02	49.13 ± 15.40	0.288
Age at Surgery, Mean ± SD	40.29 ± 15.78	42.28 ± 14.57	38.25 ± 16.83	0.196
Time to BADX (months)	6 (3–372)	12 (6–156)	6 (3–372)	0.432 ^¥^
Gender, *n* (%)				
Female	59 (57.3)	35 (67.3)	24 (47.1)	0.038 ^i^
BMI	28.45 ± 6.99	32.62 ± 5.83	22.66 ± 3.44	<0.001 *
Surgical Approach, *n* (%)				
Laparoscopic	63 (61.2)	38 (76)	25 (51)	0.010 ^i^
Comorbidities, *n* (%)				
HT	54 (52.4)	35 (74.5)	19 (43.2)	0.002 ^i^
DM	32 (31.1)	24 (51.1)	8 (18.2)	0.001 ^i^
Osteoporosis	17 (16.5)	16 (34)	1 (2.3)	<0.001 ^i^
Cancer	23 (22.3)	4 (8.5)	19 (43.2)	<0.001 ^i^
CVD	4 (3.9)	3 (6.4)	1 (2.3)	0.339
Proximal Myopathy	14 (13.6)	13 (27.7)	1 (2.3)	<0.001 ^i^
Psychiatric Disorders	7 (6.8)	5 (10.6)	2 (4.5)	0.276

Abbreviations: BADx, Bilateral Adrenalectomy; BMI, Body Mass Index; CVD, Cardiovascular Disease; DM, Diabetes Mellitus; HT, Hypertension; SD, Standard Deviation. Data are presented as mean ± SD, categorical variables are presented as *n* (%), continuous variables are reported as median (min–max); * statistical significance was determined using the independent samples *t*-test. ^i^ The chi-square test was used for categorical variables. ^¥^ *p*-values were derived from the Mann–Whitney U test.

**Table 3:** Comparison of Mortality and Long-term Complications Between Groups.

Clinical Outcome	Total (*n* = 103)	Cushing’s Group (*n* = 52)	Non-Cushing’s Group (*n* = 51)	*p*-Value
Early Mortality (≤30 days)	3 (2.9)	3 (5.8)	0	N/A
Late Mortality (>30 days)	13 (12.6)	7 (13.7)	6 (11.8)	0.767 *
Overall Mortality	16 (15.5)	10 (19.2)	6 (11.8)	0.296 *
Age at Mortality (years)	65 (34–84)	60 (44–73)	68.5 (34–84)	0.366 ^i^
Time to Mortality (months)	18 (4–372)	51 (4–372)	15.5 (7–114)	0.181 ^i^
Complications				
Adrenal Crisis	16 (15.5)	12 (23)	4 (7)	0.023 *
Nelson’s Syndrome		7 (28) **		N/A

Data are reported as median (min–max) and presented as *n* (%). * The chi-square test was used for categorical variables. ** The proportion within the Cushing’s disease subgroup is given (*n* = 25). ^i^ *p*-values were derived from the Mann–Whitney U test.

**Table 4:** Distribution of Early and Late Mortality Causes.

Number of Patients (*n*)	Etiology	Cause of Death	Timing (Early/Late)
1	CD	Intraoperative mortality (hemorrhage)	Early
1	CD	Thromboembolism	Early
1	ECS	Thromboembolism	Early
2	CD	Unknown cause	Late
1	CD	Sepsis	Late
2	AIMAH	Acute MI	Late
1	AIMAH	Unknown cause	Late
1	ECS	Unknown cause	Late
2	BAM	Sepsis	Late
2	BAM	Tumor progression	Late
1	BAM	Unknown cause	Late
1	PHEO	Adrenal crisis	Late

Abbreviations: AIMAH, ACTH-independent macronodular adrenal hyperplasia; CD, Cushing’s disease; ECS, ectopic Cushing’s syndrome; PHEO, pheochromocytoma; BAM, bilateral adrenal metastasis.

**Table 5:** Changes in Comorbidities Before and After Bilateral Adrenalectomy.

Comorbidities	CS Group (*n* = 52) Before BADx	CS Group (*n* = 52) After BADx	*p* ^i^	Non-CS Group (*n* = 51) Before BADx	Non-CS Group (*n* = 51) After BADx	*p* ^i^
DM	21 (47.7)	20 (45.5)	1.000	8 (18.2)	9 (20.5)	1.000
HT	32 (72.7)	24 (54.5)	0.057	19 (43.2)	12 (27.3)	0.092
OP	15 (34.1)	19 (43.2)	0.289	1 (2.3)	11 (25.0)	0.006
PM	13 (29.5)	2 (4.5)	<0.001	1 (2.4)	0	N/A
PD	5 (11.4)	8 (18.2)	0.375	2 (4.5)	4 (9.1)	0.625
CVD	3 (6.8)	6 (13.6)	0.250	1 (2.3)	3 (6.8)	0.500
BMI	32.69±5.94	26.31±4.98	<0.001 *	22.47±3.53	22.87±3.89	0.592 *

Abbreviations: CS, Cushing’s Syndrome; BADx, Bilateral Adrenalectomy; BMI, Body Mass Index; CVD, Cardiovascular Disease; DM, Diabetes Mellitus; HT, Hypertension; SD, Standard Deviation; OP, Osteoporosis; PD, Psychiatric Disorders; PM, Proximal Myopathy. Categorical variables are expressed as *n* (%). BMI data is presented as mean ± SD. * *p*-values were calculated using the paired samples *t*-test. ^i^ *p*-values are derived from the McNemar test for the comparison of preoperative and postoperative status. Note: Due to the retrospective nature of the study, patients with missing data for specific long-term parameters were excluded exclusively from those respective paired analyses. Consequently, the denominators (*n*) vary across different comorbidity variables.

## Data Availability

The data supporting the findings of this study are available from the corresponding author upon reasonable request.
